# Putting It Together: AIDS and the Millennium Development Goals

**DOI:** 10.1371/journal.pmed.0030455

**Published:** 2006-11-28

**Authors:** Robert Hecht, Anita Alban, Kate Taylor, Sarah Post, Nina B Andersen, Ryan Schwarz

## Abstract

Failure to halt and reverse the HIV/AIDS epidemic, say the authors, will continue to jeopardize progress on achieving a wide range of the MDGs.

## The Millennium Development Goals

One of the most important and visionary global actions of recent years was the September 2000 commitment by 189 governments worldwide to “[make] the right to development a reality for everyone and to [free] the entire human race from want” [1]. The movement to achieve sustainable reductions across all dimensions of extreme poverty has reached an unprecedented level, with efforts primarily focused on and measured against the Millennium Development Goals (MDGs; see http://www.un.org/millenniumgoals/goals.html) [2].

To date, progress toward achieving these goals has been mixed: in some regions such as East Asia, most countries have made important gains across the board and are thus “on track,” or better, for reaching many MDG targets. But in other regions, most notably in sub-Saharan Africa, a large number of countries are far behind and appear unlikely to reach, or even come close to reaching, their goals for 2015 [2]

The reasons for this are complex and often interlinked, but one stands out as a major overarching threat to development: HIV/AIDS, which kills more people than any other infectious disease and is the fourth-leading cause of death worldwide [3]. The severe health impacts of AIDS are well documented. But AIDS also affects countries' fundamental economic and social development performance, and exerts detrimental effects on many of the other MDGs. AIDS will make it difficult if not impossible for many countries to achieve their MDG targets [4].

This Policy Forum reviews the literature on the impact of AIDS on selected MDGs, and explores the links between fighting AIDS and improving other development outcomes. It also considers the implications for developing country and donor investment strategies, arguing that expanded investment in HIV prevention—especially in new prevention tools including AIDS vaccines—is essential for attaining and upholding the MDGs.

## Methodology

We examine how HIV/AIDS impedes progress toward several of the MDGs, beyond the direct target dedicated to reducing the HIV epidemic itself. Based on availability of data, we focus on the following MDGs: (1) eradicating extreme poverty and hunger; (2) achieving universal primary education; (3) reducing child mortality; (4) improving maternal health; and (5) combating malaria and tuberculosis (TB).

Our analysis draws on primary sources published over the past five years (2000–2005). The literature search, conducted between January and November 2005, relied on general searches of the PubMed database, in addition to consultations with experts from the respective fields to identify key representative peer-reviewed articles.

We give a snapshot of the effects of HIV/AIDS on each of the MDGs. The approach does not attempt to capture all the interactions among these development outcome measures. In addition, the paper is unidirectional in its analysis of the impact of AIDS: it does not try to explain, for instance, how poverty may increase transmission of and susceptibility to HIV. These relationships have been explored elsewhere [5–9].

## HIV/AIDS Increases Poverty

### Nations suffer at the macroeconomic level

Various studies, using a range of modeling techniques, argue that AIDS lowers national gross domestic product (GDP) growth by up to 1.5% annually [10]. An analysis across 80 developing countries predicts that in a “typical” African country with 20% HIV prevalence, the rate of GDP growth would be 2.6% lower each year than it would have been in the absence of AIDS. At the end of a 20-year period, GDP would be 67% lower than it would have been in the absence of AIDS at the end of a 20-year period [11].

Households face revenue losses and heavy costs. Because of high medical costs, as well as other expenses, of HIV-related illness and death, and because AIDS often kills working-age adults, the epidemic has a significant impact at the household level. Studies from Thailand and South Africa show that poverty is higher among households affected by HIV and AIDS than among unaffected families [12,13].

A recent analysis of household data from Botswana, drawing on income and expenditure surveys, suggests that HIV/AIDS can be expected to lower average income per capita by 10% over the next ten years. It also shows that the income loss is twice as large among the poorest households as it is for the overall population, suggesting that extreme poverty will become entrenched because of HIV/AIDS [14]. A similar correlation between AIDS deaths and declining household wealth was found in rural Kenyan households [15].

These effects will escalate over time. The long-term negative impact of AIDS can be expected to accelerate. Classic GDP growth models may fail to capture the negative long-term intergenerational effects of HIV/AIDS.

One analysis that models the impact of HIV/AIDS over three generations shows how AIDS could produce a progressive collapse of the economy. Declining family income forces parents to choose immediate consumption (e.g., food and medical expenditures) over long-term investments in the next generation's human capital (e.g., school fees). As a result, children of parents who die of AIDS have less human capital to pass on to their own children [16].

**Figure 1:**
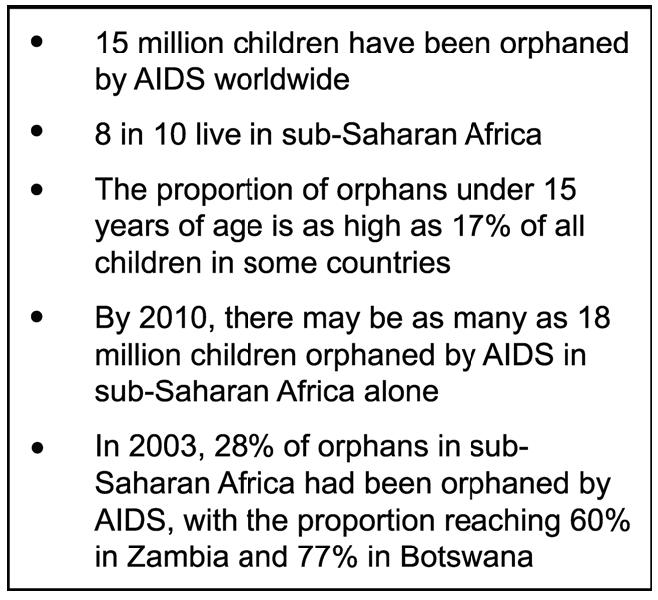
Orphans—Statistics and Predictions Data derived from [3,27,55].

The steadily increasing number of orphans in developing countries indicates how severe this problem could become (Figure 1). In South Africa, only 29% of children are expected to be living with both parents at the end of the current decade. Nearly one-fifth of children will have lost both parents [16].

## HIV/AIDS Worsens the Nutritional Status of Children

Worldwide, 53% of annual deaths among children under five are associated with malnutrition [3]. In areas with a high prevalence of HIV, progress in reducing childhood malnutrition has been mixed, and there is growing evidence of an important link between child nutrition, food security, and HIV/AIDS. In one study of 44 sub-Saharan countries, HIV prevalence was significantly negatively correlated with increasing calorie and protein consumption [8].

Some data suggest that HIV/AIDS contributes to food crises in areas where HIV prevalence is high [17]. For example, a study that examines the impact of the 2002 drought in southern Africa finds that in six affected countries, child nutrition rapidly deteriorated in the presence of high HIV prevalence. The proportion of underweight children rose from 5% to 20% in Maputo (1997–2002), with similar increases in other sub-Saharan regions. Changes were much smaller during non-drought periods and in areas with lower HIV prevalence [18].

**Figure 2:**
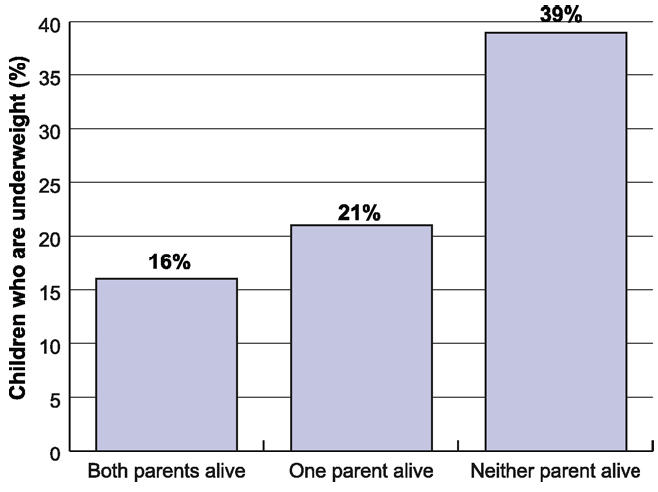
Underweight Prevalence among Children Under Four, Lesotho Figure derived from [18].

AIDS also affects child nutrition through parental mortality. Orphaned children are less likely to receive adequate nutrition. For example, in the Kagera region of Tanzania, maternal orphans lost on average one standard deviation in height, while paternal orphans' height declined one-third of a standard deviation [19]. A study from Kenya shows that weight-for-height scores in 2000 were almost 0.3 standard deviations lower for orphans [20]. In Lesotho, almost 40% of children under four who had lost both parents were underweight, compared with 16% of non-orphans (Figure 2) [18].

## AIDS Compromises Efforts to Reach Universal Primary Education

The AIDS epidemic can negatively affect both the demand for and supply of education. AIDS may prevent children from enrolling in school, or cause them to be taken out of school. It can also cause absenteeism and mortality of teachers and staff, lowering educational quality and preventing children from obtaining schooling.

AIDS reduces the demand for schooling. Children in areas affected by AIDS may drop out of school because they or their families cannot afford fees or supplies, or because their families increasingly rely on them to contribute economically to the household or to care for ill family members. In a study in the Kagera region of Tanzania, young children (seven to ten years of age) who lost their mothers had their schooling delayed, though enrollment of older children (11–14 years of age) was not affected [21]. A survey in Indonesia found that 14% of children who had lost a parent dropped out of school between ages six and ten, whereas only 7% of non-bereaved children did [22].

Another study, however, suggests that orphan status may not have a significant bearing on school enrollment [23]. The survey found larger differentials in enrollment among the 29 countries studied than between orphans and non-orphans. The authors qualify their findings by pointing out that schooling may be most affected before a sick parent dies (when time and household resources are devoted to caring for the ill adult), so the impact of parental death on enrollment may have been greater than the study found.

The theory that orphans experience disadvantages when they are not closely related to the head of the household could explain the discrepancy in these results. A review of 19 Demographic and Health Survey studies in ten African countries found a correlation between enrollment of orphans and the degree to which they were related to their guardians [24].

AIDS also hampers countries' abilities to supply education. In many countries, educational administrators face substantial challenges in replacing teachers who die. Even when replacement teachers are readily available, the death of a teacher imposes substantial costs (for temporary and permanent teacher replacement, as well as for training) on education systems [25].

**Figure 3:**
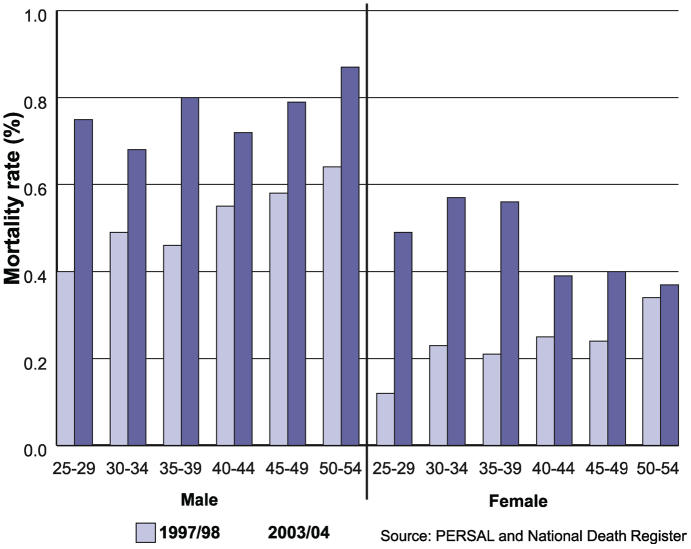
Educator Mortality Rates by Age and Gender, South Africa, 1997/1998 and 2003/2004 Figure derived from [26].

A comprehensive study of public schools in South Africa provides an overview of the attrition of educators and of mortality trends (Figure 3). Data from 1997 to 2004 show that many teachers were infected with HIV, and the total number of in-service deaths (from all causes) grew by about 30% during this period. The proportion of attrition of educators due to deaths increased from 7.0% to 17.7%, and the proportion of contract terminations attributed to medical reasons grew from 4.5% to 8.7% [26].

## AIDS Has a Negative Impact on Child Mortality

**Figure 4:**
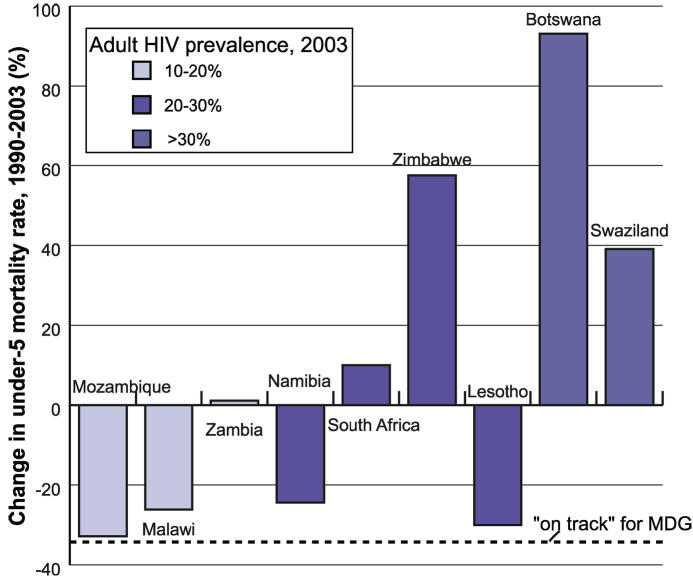
Change in Under-Five Mortality Rate in Select Countries with High HIV Prevalence, 1990–2003 Figure derived from [28].

Worldwide, approximately 10.6 million children under five died in 2003. More than 40% of these deaths occurred in Africa [27]. Many countries in Africa with a high prevalence of HIV are off track in achieving the child mortality MDG (Figure 4). In five countries that currently have adult HIV prevalence rates above 10% (Zambia, South Africa, Zimbabwe, Botswana, and Swaziland), under-five mortality not only failed to decline between 1990 and 2003, it actually increased during that period [28].

Though AIDS contributes only modestly to global child mortality [29,30], the negative effects of HIV/AIDS are significant in high-prevalence areas. For instance, one analysis estimates that by 2015, up to 90% of under-five deaths in Botswana will be directly or indirectly caused by HIV/AIDS [31]. Where the prevalence of HIV continues to increase in young women—as in many countries in sub-Saharan Africa—this effect will grow.

AIDS increases child mortality directly and indirectly. The vast majority of children living with HIV acquired the virus through perinatal transmission. And 60% of children with HIV die before their fifth birthday [32]. In this manner, AIDS directly accounted for about 570,000 child deaths in 2005 [33].

**Figure 5:**
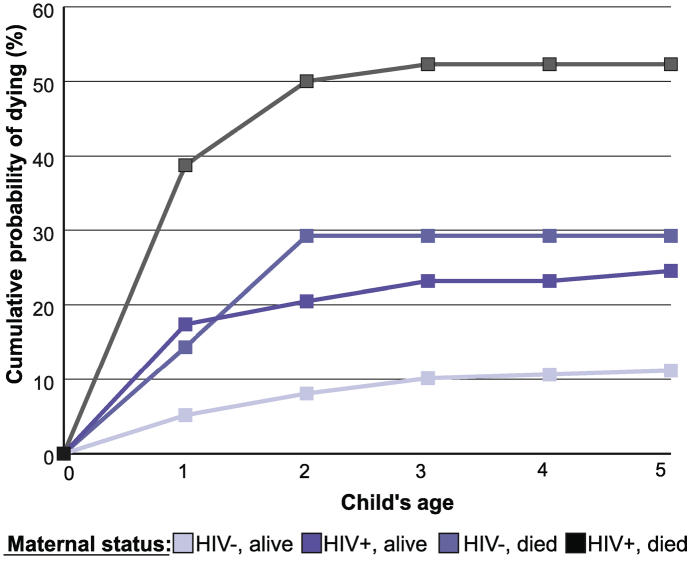
Cumulative Proportion of Children Dying by Mother's HIV Status and Survival in Uganda, 1989–2000 Figure derived from [34].

Child mortality can be attributed to AIDS even when HIV is not the direct cause of death, since children are made vulnerable to a range of economic and social “injuries” caused by their parents' illness and death. Maternal HIV status is a strong predictor of child survival, regardless of a child's HIV status [34]. Several studies have shown that children born to mothers with HIV are approximately three times more likely to die than children born to mothers not infected with HIV (Figure 5) [32,34,35]. The effect of AIDS on child mortality is increasing. An analysis of HIV-related risk of death before age five in 42 sub-Saharan African countries estimates that in 1999, HIV accounted for 7.7% of under-five mortality, up from 2% in 1990 [36]. Another study estimates that in 2002, nearly 10% of all under-five deaths in sub-Saharan Africa could be attributed to HIV/AIDS [35].

**Figure 6:**
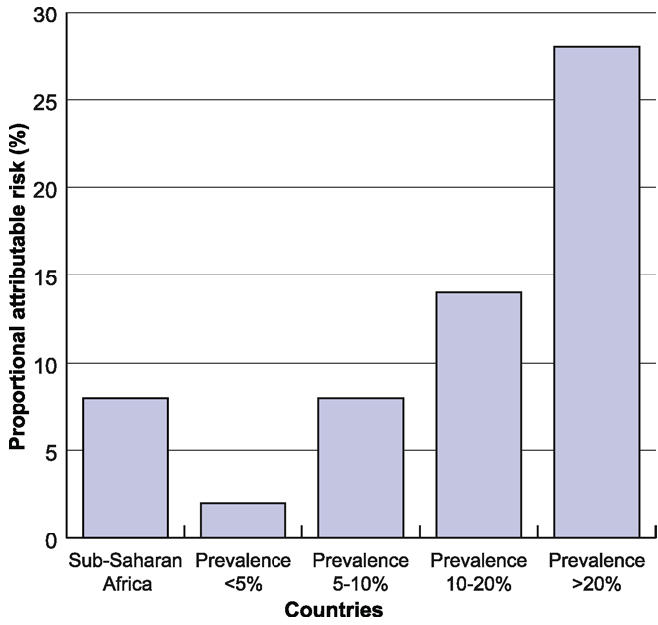
HIV-Related Population Proportional Attributable Risk of Dying before Age Five, Sub-Saharan Africa, 1999 Figure derived from [36].

When the analysis breaks down HIV-related proportional attributable child mortality for individual countries for 1999, countries with high prevalence show the effect of HIV even more strongly (Figure 6). For instance, in Botswana, Zimbabwe, and Namibia, the percentage of under-five deaths attributable to HIV/AIDS is estimated at 42.4%, 35.1%, and 26.8%, respectively [36].

## HIV/AIDS Worsens Maternal Health

Each year, more than 500,000 women die from pregnancy and childbirth-related complications, and at least 10 million suffer serious injuries or disabilities. More than 80% of these deaths occur in sub-Saharan Africa and South Asia [37].

HIV/AIDS can significantly increase maternal mortality ratios. Evidence suggests that suppressed immunity causes higher risks of prenatal and childbirth complications including miscarriage, anemia, postpartum hemorrhage, and puerperal sepsis, in addition to increasing the chances of dying from indirect causes during and after pregnancy, such as malaria or pneumonia [38].

Thus, maternal mortality ratios for women who are HIV positive can be substantially higher than for those who do not have HIV. For example, in Malawi and Zimbabwe, surveys suggest that the risk of pregnancy-related death is eight to nine times higher in women who are HIV positive. When HIV prevalence rates among pregnant women increased 10-fold between 1991 and 2001 in these countries, overall maternal mortality increased 1.9 and 2.5 times, respectively, likely as a result of this heightened risk [39]. A similar study in Rakai, Uganda, found the rate of maternal deaths of women who are HIV positive to be more than three times the rate of women who do not have HIV [40].

A study in South Africa shows that the proportion of maternal deaths due to indirect infections (including HIV) increased from 23% to 31% over the period 1998–2001, making these infections the most significant causes of maternal mortality [41]. In high-prevalence areas, this effect can substantially skew the population-wide maternal mortality ratio.

## HIV Exacerbates the Effects of Malaria

Recent studies suggest that HIV has significant effects on the incidence of malaria. HIV-induced immunodeficiency may decrease the immune response against malarial infection [42], and indeed, the risk of parasitemia and illness has been inversely correlated to CD4 cell counts [43].

HIV co-infection in areas where malaria transmission is intense primarily increases the risk of parasitemia and clinical malaria in adults [43], and clinical malarial fever in children (although the evidence supporting a link between HIV infection and clinical malarial fever is stronger in adults than in children) [44]. In unstable malaria regions, in which transmission is intermittent and unpredictable, high HIV prevalence results in significantly higher malaria morbidity and mortality [45].

Clinical studies have also shown that HIV affects the incidence of malaria and disease progression in pregnant women. Increased rates of peripheral and placental parasitemia, higher parasite densities, and higher incidence of malaria, anemia, febrile illnesses, and adverse birth outcomes have been documented with HIV and malaria co-infection [46].

Infection with HIV also affects the treatment and prophylaxis of malaria. Antimalarial therapy works best in individuals with some previous immunity to malaria, so the immune suppression associated with HIV infection may decrease the response to antimalarial therapy [47].

## HIV/AIDS Undermines Global Efforts to Control TB

The epidemics of HIV and TB are closely intertwined. Of the 40 million individuals who are HIV positive worldwide, it is estimated that nearly one-third are also infected with TB [48]. Because of HIV-related immunosuppression, individuals with HIV who carry the TB bacillus are more susceptible to active TB than carriers who are HIV negative.

The risk of acquiring TB doubles soon after HIV infection, and increases during subsequent years [49,50]. One study estimates that 9% of the 8.3 million new adult TB cases worldwide in 2000 were directly attributable to HIV [48]. In addition, HIV infection makes it harder to treat active TB successfully [49].

**Figure 7:**
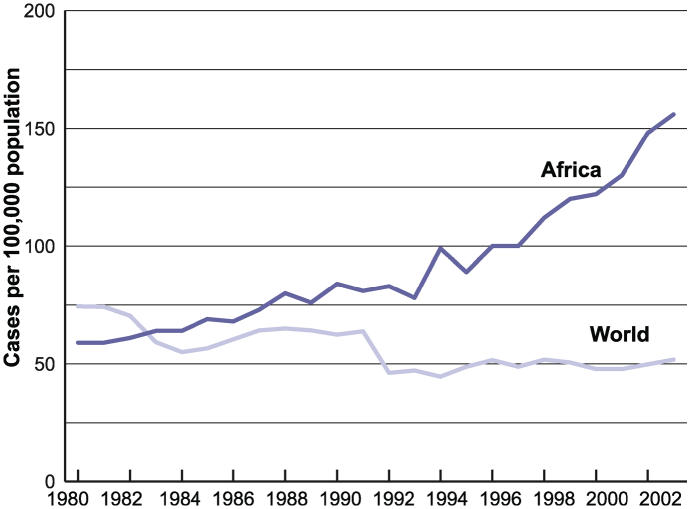
Tuberculosis Case Notification Rates, 1980–2003 Figure derived from [56].

Thus TB rates are actually increasing in high-HIV-prevalence areas of sub-Saharan Africa (Figure 7), and the spread of HIV in sub-Saharan Africa is primarily responsible for driving the number of active TB cases upward by 6% each year [48]. A recent review on progress toward reaching the MDGs argues that the AIDS epidemic represents the greatest emerging threat to TB control [51].

## Conclusion: Sustainable Strategies to Stop the AIDS Epidemic

There has been considerable progress on the global response to AIDS, with a concomitant increase in resources for the currently available range of interventions. Yet AIDS continues to ravage large parts of the world today: nearly 40 million people were living with the disease as of the end of year 2005, and more than 4 million were newly infected last year [52].

Failure to halt and reverse the AIDS epidemic will continue to jeopardize progress toward achieving a wide range of the MDGs. It will not only thwart the direct objective—stopping HIV infection and AIDS-related illness itself—but it will also undermine progress in areas as diverse as lowering poverty rates, ensuring that all children complete primary education, reducing child death rates, improving maternal health, and fighting the global malaria and TB epidemics.

From now until the 2015 MDG target date, it is essential that the delivery of existing interventions for prevention, treatment, and mitigation of the social effects of HIV be dramatically increased. The recent release of a prevention strategy from the Joint United Nations Programme on HIV/AIDS—which advocates increased funding, intersectoral efforts, and strengthening of institutional capacity—is a welcome development [53].

Yet more must be done to link HIV prevention to other development programs to ensure that they adequately address the effects of AIDS that extend beyond the domain of health to reduce the pandemic's negative effects on progress toward other social and economic goals. HIV/AIDS programs should be at the center of overall development strategies.

At the same time, it is essential to invest in the development of the new and better technologies needed for more-effective prevention, diagnosis, and treatment of HIV/AIDS. In addition to expanding treatment programs to prolong the lives of those living with HIV, the best chance of ultimately controlling the epidemic will come from new preventive technologies—notably vaccines and microbicides. Models of the impact of even partially effective vaccines with modest uptake estimate that 30–70 million individuals could be protected from HIV over 15 years of use [54].

Part of the commitment to long-term prosperity for the world's poorest countries must therefore include investing in these HIV prevention tools for the future. Governments, donors, and civil society need to increase funds for HIV prevention research and product development, and build political support, especially for the deeper involvement of developing countries. The international community must also promote expanded industry participation, a more-coordinated scientific effort, and policies and programs that accelerate the testing of novel vaccines and microbicides. Such actions are needed today and must be maintained in the future, including after 2015. The MDG targets can help assess progress and motivate political support, yet the goal of global development should be not just for 2015 but also for the entire 21st century.

## Abbreviations

GDP: gross domestic product
MDG: Millennium Development Goal
TB: tuberculosis
